# Sex work community participation in criminalized environments: a community-based cohort study of occupational health impacts in Vancouver, Canada: 2010–2019

**DOI:** 10.1186/s12939-022-01621-8

**Published:** 2022-02-09

**Authors:** Jennie Pearson, Kate Shannon, Bronwyn McBride, Andrea Krüsi, Sylvia Machat, Melissa Braschel, Shira Goldenberg

**Affiliations:** 1 Centre for Gender and Sexual Health Equity, St. Paul’s Hospital, 1081 Burrard Street, Vancouver, B.C. V6Z 1Y6 Canada; 2 Department of Medicine, University of British Columbia, St. Paul’s Hospital, 1081 Burrard Street, Vancouver, BC V6Z 1Y6 Canada; 3 Faculty of Health Sciences, Simon Fraser University, 8888 University Drive, Burnaby, BC V5A 1S6 Canada; 4 Division of Epidemiology and Biostatistics, School of Public Health, San Diego State University, San Diego, USA

**Keywords:** [sex work, Community empowerment model, Sexual health, Sex work organizing]

## Abstract

**Background:**

Sex work criminalization and occupational stigma pose barriers to sex workers’ access to support services, including community participation — engagement with sex work specific community organizing at both formalized and grassroots capacities. In light of gaps in evidence regarding impacts of community participation on sex workers’ occupational health in higher-income settings, we evaluated engagement in community participation and associations with occupational sexual health outcomes among sex workers in Vancouver, Canada.

**Methods:**

Prospective data from a community-based cohort of 943 women sex workers in Vancouver, British Columbia (2010–2019). We used logistic regression with generalised estimating equations (GEE) to model correlates of community participation, and a confounder modeling approach to examine the association of community participation on sexually transmitted infection (STI) seropositivity.

**Results:**

Among participants, 38.1% were Indigenous, 31.4% identified as women of colour (e.g., East Asian, Southeast Asian, Black) and 29.3% were im/migrants to Canada. Over a quarter (28.3%, *n* = 267) serviced in informal indoor spaces, while 38.0% (*n* = 358) serviced clients in outdoor/public and 31.4% (*n* = 296) in formal in-call spaces. 8.9% of participants reported sex work community participation at least once over the 9-year study. In multivariable GEE analysis, Indigenous (adjusted odds ratio(aOR) 1.71, 95% confidence interval (CI) 0.88–3.32) and trans women (aOR 4.69, 95%CI 2.43–9.06) had higher odds of community participation; women of colour had lower odds (aOR 0.18, 95%CI 0.06–0.57). In a multivariable GEE confounder model, community participation was independently associated with lower odds of STI seropositivity (aOR 0.66, 95% CI0.45–0.96).

**Conclusion:**

**S**ex workers who engaged in sex work community participation faced reduced odds of STI seropositivity. Building off reserach evaluating community interventions in low and middle income contexts, our study provides some of the first quantitative evidence on community participation among sex workers in Canada, and is the first to examine this in relation to sexual health outcomes. This research demonstrates the need to scale up community participation access for sex workers, via linguistically diverse community spaces, anti-stigma initiatives, and decriminalization to reduce barriers faced by racialized sex workers and support occupational health and rights for all sex workers.

## Introduction

In global contexts, sex work “community empowerment” models have been defined as community-driven approaches where sex workers “leverage collective power to address structural, behavioral and biomedical priorities” [1]. Such models have been outlined by the World Health Organization (WHO) [2], while also acknowledging that sex workers’ access to community empowerment models and sexual health services need to be supported through decriminalization [3]. Research with sex workers in certain lower and middle-income country (LMIC) contexts has shown that “community empowerment” models (collective orginizing, peer micro-loan programs e.g. have led to reductions in human immunodeficiency virus (HIV) and sexually transmitted infection (STI) incidence [4, 5], enhanced condom negotiation, reduced police and client violence [6], and improved social cohesion and financial security [7]. A review of recent evidence regarding strategies for engaging sex workers in HIV prevention and care programs found that “community empowerment” approaches where sex workers work collaboratively to address their concerns, including those beyond HIV/STIs, are those most likely to meaningfully engage sex workers [8]. In LMICs, established best practices for advancing sex workers’ occupational health and safety include “community empowerment” [4–9], yet very limited research has evaluated the impacts of “community empowerment” on sex workers’ occupational health in other contexts, such as Canada.

Sex work remains highly criminalized and stigmatized in most settings globally [10], which can have crucial negative impacts on sex workers’ health and social outcomes, including ability to organize [11–14]. In Canda, as in an increasing number of global juristictions, end-demand legislation was enacted in December 2014, which includes the criminalization of clients, third parties and sex worker collectivization [15, 16]. The continued surveillance of sex workers under this model of criminalization has been found to rush service negotiation and screening, increasing risk of violence and HIV/STIs [17] and impeding sex workers’ access to sexual health and other support services [1, 10, 18, 19]. Services not informed by the unique and diverse needs of sex workers may also reinforce stigma, rather than offer meaningful and impactful support and services [8, 20]. The substantial health, social and economic inequities experienced by sex workers highlights the need to examnine sex work community participation and how it may promote better occupational health and safety. As well, ongoing criminalization, stigma, and a lack of funding often hinder the collectivisation of sex workers and further expansion of community supports [21]. Due to such limitations, existing supports may not be accessible or suited to diverse sex work communities, and these gaps may exacerbate existing health inequities faced by sex workers. As such, research is needed to understand sex workers’ access and engagement with community participation, including how such participation may be disproportionately available to certain groups, and how community participation impacts sex workers’ sexual health.

While less literature exists on the outcomes of sex worker-led initiatives in higher-income settings such as Canada, communities in Canada have demonstrated “decades of creative, collaborative, and revolutionary community-building, outreach and support, awareness raising, rabble-rousing, and legislative work” [14]. Such organizing is described in this study as community participation (CP) — defined as having participated or engaged with any sex work community organizing or peer-based initiatives at both formalized and grassroots capacities. Throughout Metro Vancouver, but largely concentrated in the city’s Downtown East Side (DTES), sex worker-specific services provide critical opportunities for community participation via peer support and education programs, outreach-based supports, drop-in spaces, and low-threshold occupational health and safety and harm reduction supplies (e.g., bad date sheets, condoms) [22, 23].

The COVID-19 pandemic has brought into stark focus the urgent need to address the unmet occupational health priorities of sex workers in Vancouver and elsewhere, and has further highlighted the severe impacts of stigma and criminalization on sex workers’ access to structural supports [24], as well as the underfunding of essential community services [25]. As such, there is a particularly timely need examine the outcomes of community participation, and existing barriers. Inspired by the body of literature on community participation initiatives for sex workers in LMICs, our objectives were to (1) identify correlates of community participation among sex workers in Vancouver, Canada, and (2) examine the independent association between community participation and STI seropositivity.

## Methods

### Study design

Data were drawn from a community-based open longitudinal cohort, An Evaluation of Sex Workers Health Access (AESHA), which initiated recruitment in 2010. This study was developed based on substantial community collaborations with sex work agencies since 2005 [26], is monitored by a Community Advisory Board of 15+ community agencies, and has included experiential staff (current/former sex workers) since inception. Eligibility includes identifying as a cis or trans woman, being 14 years old or older at enrolment, having exchanged sex for money within the last 30 days, and providing written informed consent. To address the challenges of recruiting stigmatized and hidden populations such as sex workers, time-location sampling was used to recruit participants through daytime and late-night (9 pm–2 am) outreach to outdoor/public sex work locations (e.g., streets, alleys) and indoor sex work venues (e.g., massage parlors, micro-brothels, and out-call locations) across Metro Vancouver, BC. In addition, online recruitment is used to reach sex workers working through online solicitation spaces. Indoor sex work venues and outdoor solicitation spaces (‘strolls’) are identified through community mapping and are updated regularly by the outreach team.

Following informed consent, sex workers completed interview-administered questionnaires at baseline and semi-annually. Interviews were conducted at study offices in Metro Vancouver or a confidential space of their choice (e.g., home, work). The questionnaire is administered by a trained interviewer and data are securely collected and managed using REDCap electronic data capture tools [27, 28]. The questionnaire includes questions related to individual socio-demographic characteristics (e.g., age, sexual and gender identity and orientation, physical and mental health, patterns of substance use), sex work history and patterns, and structural factors [29]. Questions on structural factors included education, racialization, physical and sexual workplace violence, work environment, criminalization, interactions with police, community participation, and access to health and social services (e.g., unmet health needs; barriers to accessing diverse health and social services). The questionnaire is updated regularly in order to capture emerging and changing priorities and needs within the community. All participants received 40 Canadian Dollars (CAD) at each semi-annual visit for their time, expertise and travel.

### Study variables

#### Sex work community participation variable

Drawing on existing measures of sex work ‘community empowerment ’[30], our primary exposure variable was a time-updated measure of “sex work community participation” (CP), defined as listing a sex work organization in response to the question “In the last 6 months, have you participated, worked, or volunteered with any community organizations or peer-based initiatives? If yes, which organizations?” Sex work organizations were Metro Vancouver organizations which provide services to sex workers, such as health services and drop-in spaces, many of which are sex worker-led or have implemented peer staff models. Grassroots forms of sex work community participation, such as “spotting” for other sex workers (e.g., tracking client data) and street cleanup, were also captured.

#### STI Seropositivity outcome

Our primary outcome variable was “STI seropositivity”, which was a time-updated measure defined as a positive STI test (syphilis, chlamydia, or gonorrhea) at each study visit. Following extensive pre-test counselling, participants receive voluntary testing by a project nurse and are offered referrals or STI treatment onsite, if needed, regardless of enrolment in the study. At each semi-annual visit for voluntary sexual health testing, urine samples were collected for gonorrhea and chlamydia, and blood was drawn for syphilis. Syphilis was tested using the rapid plasma reagin (RPR) (97.2% Se; 94.1% Sp) and the *Treponema pallidum* hemagglutinin assay (TPHA) for all samples with positive RPRs. RPR titers≥1:8 was considered indicative of active infection in the absence of treatment. All participants received post-test counselling. Treatment was provided by a project nurse onsite for symptomatic STIs.

#### Other explanatory variables

Other explanatory variables of interest were selected a priori based on literature related to “community empowerment” and sex workers’ health and safety. For time-fixed socio-demographic variables, we measured race and Indigenous identity to examine the effects of racism defined as Indigenous (inclusive of First Nations, Métis, or Inuit), Women of Colour (e.g., Black, East Asian, Southeast Asian) vs white. Given the low proportion of participants who identified as Black in our sample, we combined Black and Women of Colour (WOC) for analyses to examine effects of racialized community identities. We also measured high school attainment, im/migration to Canada, sexual minority (lesbian, bisexual, asexual, queer vs. heterosexual), trans women (including transgender women, transexual women and other transfeminine identities- vs cisgender women). Other individual variables were time-updated at each semi-annual study visit, including age (continuous, in years) and years in sex work (continuous). Time-updated variables capturing experiences in the last six months included inconsistent condom use with clients (i.e., using condoms less than 100% of the time for vaginal or anal sex with clients), any non-injection drug use (excluding alcohol and cannabis) and any injection drug use.

Time-updated, structural variables included average weekly income from servicing clients ($CAD), any barriers to receiving health care (e.g., lack of availability, language barriers, poor treatment by health care providers, etc.). We assessed recent (last six months) violence experiences including rape or physical violence by aggressor posing as a client; negative police encounters while working; spent time in jail overnight or longer; and primary place of service, including outdoor/public (e.g. street, public washroom, car, tent), informal indoor (e.g. sauna/steam bath, bar/nightclub, own or client’s place of residence), or formal in-call (e.g. massage/beauty parlor, micro-brothel). This variable included a category for no recent sex work, as not all participants do sex work at every semi-annual visit. Two variables captured place of residence: living in Vancouver’s Downtown Eastside (DTES) and living within the City of Vancouver. The DTES represents an inner-city neighborhood within the City of Vancouver’s downtown core where community organizations and low-barrier services are heavily concentrated.

#### Statistical analyses

Baseline descriptive statistics for individual and structural characteristics were calculated as frequencies and proportions for categorical variables and measures of central tendencies (i.e., mean, median, interquartile range (IQR)) for continuous variables. These were stratified by the primary dependent variable of interest and compared using Pearson’s chi-square test for categorical variables (in the case of small cell counts, Fisher’s exact test was used in place of Pearson’s chi-square) and the Wilcoxon rank-sum test for continuous variables.

Bivariate and multivariable analyses used logistic regression with generalized estimating equations (GEE) and an exchangeable correlation matrix to account for repeated measurements amongst participants over time [31]. Bivariate analyses examined associations with community participation over the study period. Explanatory variables significantly associated with community participation at *p* < 0.10 were considered for inclusion in the multivariable explanatory model. The multivariable model with the best overall fit, indicated by the lowest quasi-likelihood under the independence model criterion, was determined using a backward selection process. Finally, we developed a separate multivariable GEE confounder model to identify the independent association of community participation on STI seropositivity. All variables from the full explanatory model were considered as potential confounders and included sexual minority, trans identity, racialization, im/migration status, living in the City of Vancouver, rape, and weekly income from servicing clients. Statistical analyses were performed in SAS version 9.4 (SAS, Cary, NC), and all *p*-values are two-sided.

## Results

Analyses included 6132 observations on 943 participants from January 2010–February 2019, and participants contributed a median of 5 study visits (IQR = 2–11). At baseline, 29 (3.1%) participants reported having participated, worked, or volunteered with a sex work organization in the last six months, and 84 (8.9%) reported community participation at least once throughout the study. Participants who engaged in community participation at baseline reported an average of 4 h of participation per week (IQR = 2–8).

At baseline (Table 1), participants’ median age was 35 (IQR 28–42), with a median weekly income of $500 CAD from servicing clients (IQR $250–1000 CAD). More than a third (38.1%, *n* = 359) of participants were Indigenous, 31.4% (*n* = 296) were women of colour (primarily South or East Asian, with a small number of participants identifying as Black) and 29.3% (*n* = 276) were im/migrants to Canada. At baseline, zero participants who were im/migrants or women of colour reported sex work community participation in the last six months. Approximately one-third (31.9%, *n* = 301) identified as a sexual minority and 6.2% (*n* = 58) identified as trans women. Over a quarter (28.3%, *n* = 267) serviced in informal indoor spaces, while 38.0% (*n* = 358) serviced clients in outdoor/public and 31.4% (*n* = 296) in formal in-call spaces. Almost half (40.6%, *n* = 383) reported using injection drugs in the last six months, and 6.9% (*n* = 65). Two thirds (66.9%, *n* = 631) reported living within the City of Vancouver.

**Table 1:** Baseline individual and structural characteristics of sex workers in Metro Vancouver, Canada, stratified by recent participation in sex work community organizing (*n* = 943), AESHA 2010–2019

Characteristic	Total (%) (***n*** = 943)	Participated in sex work community organizing^c^		***p*** - value
		Yes (***%***) (***n*** = 29)	No (%) (***n*** = 914)	
***Personal and Interpersonal Factors***				
Age (median, IQR)	35 (28–42)	42 (32–45)	35 (28–42)	0.007
Years in sex work (median, IQR)	10 (3–19)	16 (9–32)	10 (2–18)	< 0.001
Sexual Minority (LGBQ2S+)^a^	301 (31.9)	14 (48.3)	287 (31.4)	0.056
Trans identity^b^	58 (6.2)	8 (27.6)	50 (5.5)	< 0.001
Inconsistent condom use with clients^c^	166 (17.6)	5 (17.2)	161 (17.6)	0.931
STI seropositivity	98 (10.4)	2 (6.9)	96 (10.5)	1.000
Injection drug use^c^	383 (40.6)	13 (44.8)	370 (40.5)	0.639
Non-injection drug use^c^	626 (66.4)	25 (86.2)	601 (65.8)	0.022
***Structural Factors***				
High School Attainment	525 (55.7)	18 (62.1)	507 (55.8)	0.571
*Racialization*				
White	288 (30.5)	7 (24.1)	281 (30.7)	
Indigenous	359 (38.1)	22 (75.9)	337 (36.9)	
Woman of Colour	296 (31.4)	0 (0.0)	296 (32.4)	< 0.001
Im/migrated to Canada	276 (29.3)	0 (0.0)	276 (30.2)	< 0.001
Living in Vancouver’s Downtown East side	291 (30.9)	15 (51.7)	276 (30.2)	0.017
Living in the City of Vancouver	631 (66.9)	28 (96.6)	603 (66.0)	0.001
Experienced any barriers to receiving health care^c^	626 (66.4)	18 (62.1)	608 (66.5)	0.617
Any negative police encounters while working^c^	330 (35.0)	11 (37.9)	319 (34.9)	0.743
In jail overnight or longer^c^	127 (13.5)	4 (13.8)	123 (13.5)	1.000
Experienced Rape^c^	65 (6.9)	4 (13.8)	61 (6.7)	0.139
Average weekly income from servicing clients ($ CAD)^c^ (median, IQR)	500 (250–1000)	400 (250–675)	500 (250–1000)	0.075
*Primary place serving clients*^†^				
Outdoor or public space	358 (38.0)	10 (34.5)	348 (38.1)	
Informal indoor space	267 (28.3)	16 (55.2)	251 (27.5)	
In-call sex work venue	296 (31.4)	3 (10.3)	293 (32.1)	0.007

All data refer to *n* (%) of participants, unless otherwise specified

^a^Gay, lesbian, bisexual, two spirit, asexual, queer, other

^b^Trans women - including transgender women, transexual women and other transfeminine identities- vs cisgender women

^c^In the last 6 months

In unadjusted GEE explanatory analysis; Indigenous (odds ratio (OR) 2.40, 95% CI 1.26–4.57) sexual minority (OR 2.27, 95% CI 1.31–3.95), and trans women (OR 7.84, 95% CI 4.23–14.52) sex workers and those living within local policing jurisdiction compared to federal RCMP (OR 2.56, 95% CI 1.07–6.10), and who experienced recent client violence (OR 1.89, 95% CI 1.22–2.94) had higher odds of community participation, whereas women of colour (OR 0.14, 95% CI 0.04–0.44). and sex workers with higher weekly sex work income (OR 0.98 per $100 CAD, 95% CI 0.96 to 1.00) had lower odds (Table 2).

**Table 2:** Correlates of recent sex work community participation among sex workers in Metro Vancouver, Canada (*n* = 943), AESHA 2010–2019

	Unadjusted		Adjusted	
Characteristic	Odds Ratio (95% CI)	***p -*** value	Odds Ratio (95% CI)	***p -*** value
Socio-demographic Factors				
Age (per year older)	1.01 (0.97–1.06)	0.583		
Years in sex work (continuous)	1.018 (0.99–1.05)	0.285		
Sexual Minority **(LGBQ2S+)**^a^	2.27 (1.31–3.95)	0.004	d	
Trans identity^b^	7.84 (4.23–14.52)	< 0.001	4.69 (2.43–9.06)	< 0.001
Inconsistent condom use with clients^c^	0.91 (0.58–1.42)	0.681		
Injection drug use^c^	1.12 (0.74–1.70)	0. 598		
Non-injection drug use^c^	1.34 (0.68–2.64)	0.402		
***Structural Factors***				
*Racialization*				
White	Ref			
Indigenous	2.40 (1.26–4.57)	0.008	1.71 (0.88–3.32)	0.112
Woman of Colour	0.14 (0.04–0.44)	0.001	0.18 (0.06–0.57)	0.004
Im/migrated to Canada	0.08 (0.03–0.20)	< 0.001	d	
Living in DTES	1.37 (0.87–2.15)	0.174		
Living in the City of Vancouver	2.56 (1.07–6.10)	0.035	2.18 (0.95–5.01)	0.065
Experienced any barriers to receiving health care^c^	0.95 (0.70–1.28)	0.712		
Criminalization				
Any negative police encounters while working^c^	1.12 (0.72–1.75)	0.611		
In jail overnight or longer^c^	1.17 (0.76–1.82)	0.474		
Forced to have sex with aggressor posing as client^c^	1.89 (1.22–2.94)	0.005	2.06 (1.24–3.42)	0.005
Average weekly income from servicing clients (per $100 CAD)^***c***^	0.98 (0.96–1.00)	0.099	0.98 (0.95–1.00)	0.087
*Primary place servicing clients*^*c*^				
Outdoor or public space	Ref			
Informal indoor space	1.03 (0.72–1.46)	0.878		
In-call sex work venue	0.58 (0.30–1.12)	0.103		
No recent sex work	0.53 (0.25–1.11)	0.092		

^a^Gay, lesbian, bisexual, two spirit, asexual, queer, other

^b^Trans women - including transgender women, transexual women and other transfeminine identities- vs cisgender women

^c^Time updated to capture events in the last six months

^d^Variable was included in multivariable analysis but was not retained in the best fitting model

In multivariable GEE explanatory analysis (Table 2), Indigenous (adjusted odds ratio (aOR) 1.71, 95% CI 0.88–0.32) and trans women (aOR 4.69, 95% CI 2.43–9.06) had higher odds of community participation, whereas other WOC had lower odds (aOR 0.18, 95% CI 0.06–0.57). In a multivariable GEE confounder model, community participation retained a significant association with reduced odds of STI seropositivity (aOR 0.66, 95% CI 0.45–0.96) after adjusting for confounders (Table 3).

**Table 3:** Association Between Recent Sex Work Community Participation And STI Seropositivity Among Sex Workers In Vancouver, Bc, [2010–2019] (*N* = 943)

	Outcome: STI seropositivity Unadjusted Odds Ratio (95% CI) Adjusted Odds Ratio (95% CI)	
Participated in sex work community organizing^a^ (yes vs. no)	0.69 (0.50–0.94)	0.66 (0.45–0.96)*

^a^Time updated to capture events in the last 6 months

* GEE confounder model adjusted for hypothesized confounders, including racialization, average weekly income from servicing clients^a^, living in the City of Vancouver, sexual minority, trans identity, im/migration to Canada, and experienced rape_a_

## Discussion

We found very low levels of reported community participation (3.1% at baseline and 8.9% during the entire 9-year observation period) while revealing varying levels of access amongst sub-communities of sex workers. Our findings show that sex workers who identified as Indigenous and as trans women, and those living within the City of Vancouver had higher odds of community participation, while women of colour and im/migrant sex workers had lower odds of community participation, suggesting that they may experience barriers due to compounding criminalization as both sex workers and racialized im/migrant workers. Sex workers who engaged in community participation demonstrated reduced odds of STI seropositivity, highlighting the benefits of community participation in sex workers’ occupational health and safety.

Our results show increased engagement with community participation among Indigenous, trans women, lower-income sex workers and those living within the city of Vancouver. This may be attributed to the concentration of sex worker-specific services situated in the inner-city community especially, as well as Indigenous and trans women sex workers’ involvement and leadership in creating sex worker-led support services within downtown Vancouver, such as Providing Advocacy Counselling and Education (PACE) Society and Sex Workers United Against Violence (SWUAV) [14]. More than half (51.7%) of sex workers who reported community participation in the last six months at baseline also reported living in the Downtown East Side (DTES), a neighbourhood within the City of Vancouver characterized by both social and economic inequities as well as significant community organizing and low-threshold services. Indigenous women are also over-represented within the DTES and its sex work community [32]. Though facing ongoing inequities, policing and gentrification, organizers within the DTES have been successful in scaling up sex work-specific supports that reach local and neighbouring community members. Throughout the COVID-19 pandemic as well, sex work organizations have been highly successful in pivoting their services in order to reach diverse communities of sex workers [33, 34].

Our study found a near-total lack of sex work community participation among WOC and im/migrant sex workers, who may face unique barriers to community participation due to compounding criminalization, language barriers, socio-spatial limitations (proximity to organizations, access to transportation, e.g.), cultural barriers and exacerbated occupational stigma [20, 21]. More punitive policing measures and increased surveillance of indoor venues, may also limit racialized and im/migrant sex workers’ ability to access sex work-specific services. For example, ongoing and disproportionate criminalization of racialized im/migrant sex workers has been found to be independently linked to reduced access to health services [35]. Im/migrant sex workers may be hesitant to access services, including sex worker-specific services, in fear of possible legal ramifications. Research assessing the needs of Asian, im/migrant sex workers in Toronto, Canada found that just over a half of participants accessed any social services, and less then 5% reported accessing sex work-specific supports. Not wanting to disclose immigration or sex worker identity and language were noted as the main barriers to access [20]. Additionally, research within Metro Vancouver has found that racialized and im/migrant sex workers are more likely to work in managed, indoor work venues and frequently work amongst other sex workers [31]. This specific work environment may also offer similar benefits of community participation not captured in this study.

Lastly, the findings of the current study echo research in LMICs which has shown that sex work community participation and social cohesion among sex workers, including work from Songachi in India [11, 12] and sex work organizing in Brazil [36], plays a critical role in supporting sex workers’ ability to access and engage in occupational health and safety resources, such as client condom negotiation and access to sexual health testing. Our study builds on this body of work by providing some of the first quantitative data on sex workers’ engagement and sexual health outcomes associated with community participation in North America. Additionally, whereas much previous research has evaluated ongoing community mobilization interventions, our study more broadly addressed sex workers’ participation in various sex worker-specific community participation initiatives across Metro Vancouver, and provides opportunities to understand both access and outcomes of these diverse forms of participation. Our research builds on previous work that has shown links between uptake of sex worker-led drop-in services and utilization of sexual and reproductive health services among marginalized sex workers [22]. Additionally, our findings are supported by previous work which found that community social cohesion among sex workers (ability of sex workers to work together) was associated with enhanced ability to negotiate terms of service and reduced client condom refusal [21, 23]. Our results build on this work, adding unique insights regarding the relationship between community participation and STI seropositivity, highlighting the crucial role of community in sex workers’ occupational health and safety, and identifying sub-groups of sex workers for whom community participation may not be accessible.

In our study, only 8.9% of sex workers engaged in community participation over a nine-year period, and women of colour and im/migrant sex workers faced the lowest engagement in community participation. Global reviews of sex work “community empowerment” [8] have shown that despite robust evidence for scaling up comprehensive community participation models, there remains limited programming and focus on meaningful engagement with diverse sex workers. Criminalized legislative environments have been found to exacerbate barriers to accessing health and community-led services proven to be key contributors of better health outcomes [21, 31]. Availability of funding for human rights-based approaches to sex work organizing has historically been hindered in North America and elsewhere by harmful anti-trafficking rhetoric, and within the context of Metro Vancouver, sex work organizations have been recommended to adopt abolitionist approaches to their services [37].

Current research supports ongoing call for evidence-based interventions to enhance sex workers’ occupational health and safety that address criminalization and structural inequities, including through community participation [10, 17] – these calls remain more urgent than ever amidst the COVID-19 pandemic, as sex work communities have reported important unmet social, economic, and health-related needs. Although the current study identified an important association between community participation and reduced odds of STI seropositivity, interventions focusing narrowly on HIV/STI prevention and care may undermine sex workers’ broader priorities beyond sexual health [8]. This research aligns with calls for evidence for scaling-up holistic, and comprehensive, multi-component community participation programs by and for sex workers, in conjunction with decriminalization, as has recommended by Amnesty International and community-based recommendations [12].

### Strengths and limitations

Strengths of this study include its longitudinal nature, strong community collaborations, and large, diverse sample. Aside from STI seropositivity, all data are self-reported. As with most research involving stigmatised populations, there is potential for social desirability and recall bias; our community-based and experiential team, training in non-stigmatising interview techniques, and community collaborations are designed to mitigate this. Additionally, due to the various health and safety inequities faced by sex workers, there is chance of loss to follow-up. The weekly time-location outreach conducted by our interview team, and frequent check-ins with participants helps minimize loss to follow up. This research cannot infer causality and findings may not be fully generalizable to other sex worker populations, including Black sex workers, who may be underrepresented. However, the mapping of working areas and time–location sampling likely helped ensure a representative sample and minimize selection bias. Broad response options included in the questionnaire may pose limits to participants’ ability to identify certain forms of “community participation”. Further research is needed to capture more nuanced forms of sex work community participation.

## Conclusions

In summary, the present results indicate an independent association between sex work community participation, defined as engagement with sex work community organizing, and sex workers’ reduced odds of STI seropositivity, being among the first studies within Canadian contexts to mirror results found in LMI settings [4–9]. However, we also find that barriers to community participation remain for many, particularly WOC, im/migrant sex workers. This research demonstrates the need to scale up community-led initiatives for sex workers that are low barrier, non-stigmatizing, culturally safe, anti-racist, linguistically diverse and that reach sex workers in more diverse geographical areas. There is need for greater funding for sex work community participation that allows flexible granting mechanisms and support a human rights based approach. Furthermore, decriminalization of sex work, including im/migrant sex work, and sex workers’ equal access to structural supports are important steps toward reducing barriers to critical health and social services.

## Data Availability

The datasets generated and/or analysed during the current study are not publicly available but are available from the corresponding author on reasonable request.

## Abbreviations

CP: Community Participation
HIV: Human Immunodeficiency Virus
STI: Sexually Transmitted Infection
LMI: Lower and Middle Income
WOC: Women of Colour
AESHA: An Evaluation of Sex Workers’ Health Access
DTES: Downtown Eastside
